# *Stuff you think you can handle as a parent and stuff you can’t’*. Understanding parental health‐seeking behaviour when accessing unscheduled care: A qualitative study

**DOI:** 10.1111/hex.13305

**Published:** 2021-07-06

**Authors:** Ciara Conlon, Emma Nicholson, Aoife De Brún, Therese McDonnell, Eilish McAuliffe

**Affiliations:** ^1^ UCD Centre for Interdisciplinary Research Education and Innovation in Health Systems UCD School of Nursing Midwifery & Health Systems University College Dublin Dublin Ireland

**Keywords:** emergency care, general practitioner, health‐seeking behaviour, paediatric health care, parental decision making, unscheduled health care

## Abstract

**Background:**

Unscheduled health care constitutes a significant proportion of health‐care utilization. Parental decision making when accessing unscheduled care for their children is multifaceted and must be better understood to inform policy and practice.

**Design:**

Nineteen semi‐structured interviews and one focus group (n = 4) with parents of children younger than twelve in Ireland were conducted. Participants had accessed unscheduled care for their children in the past. Data were thematically analysed.

**Results:**

Parents accessed unscheduled care for their children after reaching capacity to manage the child's health themselves. This was informed by factors such as parental experience, perceived urgency and need for reassurance. Parents considered the necessity to access care and situated their health‐seeking behaviour within a framework of ‘appropriateness’. Where parents sought unscheduled care was largely determined by timely access, and inability to secure a general practitioner (GP) appointment often led parents to access other services. Parents expressed a need for more support in navigating unscheduled care options.

**Conclusions:**

Better resources to educate and support parents are required, and structural issues, such as accessibility to GPs, need to be addressed to enable parents to better navigate the unscheduled health system and manage their children's health. The discourse around ‘appropriate’ and ‘inappropriate’ access to health care has permeated parental decision making when accessing unscheduled health care for their children. What constitutes appropriate access should be examined, and a shift away from this framing of health‐seeking behaviour may be warranted.

**Patient or Public Contribution:**

There was no explicit patient or public involvement. All authors hold experience as users of the health system.

## 1 BACKGROUND

Unscheduled health care is non‐routine care accessed in an unplanned manner, ranging across primary care, emergency departments (EDs), hospital admission and specialized hospital support.^1^ In seeking unscheduled care, patients may access multiple services, both in and out of normal working hours.^2^, ^3^ It is commonly delivered by general practitioners (GPs), out‐of‐hour GP services (OOHs), urgent care centres, minor injury units, ambulance services and EDs, although models vary across countries and health systems. Unscheduled care represents a sizeable proportion of health‐care utilization. One study in England spanning five years found that 16% of the population accessed some form of unscheduled care in a four‐week period, with parents on behalf of their children younger than five twice as likely to access such care.^3^ Access to unscheduled health care is unpredictable and presents challenges to service planners, although temporal patterns of higher demand, such as early in the week and during the winter season, have been identified.^4^

The model of unscheduled care is ambiguously configured on a hierarchy of need and urgency with unspecific boundaries between services; thus, for a multitude of reasons, users may fail to distinguish and utilize services in the manner intended by policymakers.^5^ Internationally, the rise in demand for unscheduled care, particularly ED visits, has garnered attention, with many calling attention to the implications, such as overcrowding and longer waiting times.^6^, ^7^ In response, health policies have focused on reducing ED visits,^8^, ^9^ in particular visits deemed ‘inappropriate’.^10^, ^11^ Studies have suggested up to 40% of presentations to EDs are non‐urgent,^12^ and parents accessing care for their children have been identified as frequently presenting to EDs with conditions deemed treatable in primary care. ^9^, ^11^ However, estimates vary considerably^10^ and concerns have been raised about reliability and standardization of categorizing urgency.^7^ The focus on non‐urgent ED use, exacerbated by unrelenting pressure on emergency services, has resulted in health‐care utilization being dichotomized into ‘appropriate’ or ‘inappropriate’ categories. This framing creates a discourse imbued with moral judgements of health‐seeking behaviour,^5^, ^13^ leading some to call for the underlying assumptions of what constitutes appropriate access to health care to be examined.^10^, ^14^

Assumptions of inappropriate care delivered in the ED are not informed by evidence of health‐seeking behaviour, namely what leads patients with low‐urgency conditions to the ED for care.^10^ Research into health‐seeking behaviour has shown that clinically unnecessary ED visits are often driven by multiple and concurrent mechanisms.^13^ Factors that determine when and where unscheduled care is accessed are relevant to the individual patient, the health‐care organization and the wider social context.^1^ The influence of health system factors may vary extensively,^1^ with level of access to primary services having a marked impact.^9^, ^15^ Geographical distance to the ED and whether or not upfront payment is required have been found to influence patient's decision making when accessing higher acuity services ^2^, ^16^, ^17^ Increased use of unscheduled care at the ED is associated with demographic factors such as lower socio‐economic status, driven by health inequality and lack of access to health insurance.^12^, ^14^

Parental decision making has additional complexity as potential consequences of making decisions regarding their children's health may create anxiety.^13^ Parents report a need to minimize risk and seek reassurance, which can be initiated by the perceived urgency of the child's condition.^13^, ^18^ Parental health literacy influences how parents seek health care for their children, and impacts their ability to navigate complex unscheduled health systems.^18^, ^19^ Timely access, convenience, preference based on past experience and confidence in primary care providers have also been found to influence parents when seeking unscheduled care for their children.^13^, ^16^, ^17^, ^18^

Given the multifaceted nature of health‐seeking behaviour, understanding the decision making of patients is crucial to inform successful policy and practice.^7^ Inquiries into unscheduled care use typically isolate one component, giving an incomplete and limited understanding of the realities of the complex decision‐making process that parents engage in.^2^, ^3^, ^18^ A multiplicity of services offer unscheduled care; thus, conceptualizing it as a system of care provision may assist in assessing the functioning of care delivery and further understanding on how and why users utilize unscheduled care. ^3^, ^18^, ^20^ Utilizing this systematic concept of unscheduled care provision, this research sought to capture parents’ experiences and decision making when seeking unscheduled health care for their children.

## 2 METHODS

This study adopted a qualitative approach to capture peoples’ lived experiences and gain a more in‐depth insight into parental decision making and the experience of parents when seeking unscheduled health care for their children.

### 2.1 Setting

Unscheduled health care in Ireland is largely provided by GPs, OOH GP services, EDs and local injury units (LIUs). A visit to a GP in Ireland costs on average €51,^21^ while attending the ED costs €100. However, children younger than six qualify for a GP visit card, which entitles the holder to free at point of delivery GP and OOH GP services, and a referral to the ED from general practice waives the €100 fee. Additionally, access to health care at no charge is provided to those on low income or with a qualifying medical condition through the General Medical Service (GMS) scheme, irrespective of age. Means‐tested GP visit cards, with an income threshold above that of the GMS scheme, also entitle the holder to free GP care. LIUs treat minor injuries at a cost of €75 per visit, with access at no charge to those with a GMS card or GP referral, though most LIUs do not provide treatment to children younger than five years.

### 2.2 Recruitment

The sampling criteria included all parents of children younger than 12 living in Ireland. Convenience sampling was employed by approaching potential participants through parent support groups, childcare facilities and children's sports activities. Managers or gatekeepers of these groups/facilities were contacted to request facilitation of recruitment, after which a researcher attended groups/ facilities to distribute information sheets. Those interested in participating had the option to provide their contact details or contact the researcher later. In some cases, gatekeepers identified willing participants by providing potential participants with the information sheet, and forwarded the contact details of those interested in participating to the research team. Written consent was obtained from participants before interviews commenced.

### 2.3 Data collection

One focus group (n = 4) and 19 semi‐structured one‐on‐one interviews were carried out between October 2019 and March 2020. Participants were asked whether they preferred to attend a focus group or an interview. Several participants who were recruited from one location (a university childcare facility) indicated interest in a focus group, which was then arranged at a convenient time. The smaller number of participants recruited from each of the other sites made this option impractical; thus, only one focus group was carried out. Focus groups bring together participants with the same frame of reference, which, through the introduction of a group dynamic, can facilitate the understanding on participants’ attitudes and patterns of thought, and reveal dominant norms and values.^22^, ^23^ One‐to‐one interviews elicit more in‐depth data on the participant's experience and their decision‐making process. Critical incident technique (CIT) was utilized in interviews to further understanding of the participant's decision‐making process.^24^ CIT focuses the participant on their memory of a particular event, in this case, an incident when their child fell sick and they could no longer manage the situation themselves. The interviewer then asked probing questions to delve deeper into this recalled event to apprehend the cognitions and behaviours of the participant during that event. If they could not recall a specific incident, they were asked about their general experiences of seeking unscheduled care. Both the interviews and the focus group took place at a time and place convenient for the participant. Locations included coffee shops, hotels, the university the researchers worked in or the participant's home and lasted approx. 30‐60 minutes. The focus group lasted one hour. One researcher (CC) carried out the interviews and focus group. The interviewer collected demographic data, such as age, family status and education, using a questionnaire before utilizing an interview guide (see Appendix 1), which was informed by a systematic review carried out prior to data collection.^18^ The interview guide was altered for the focus group (see Appendix 2). Interviews ceased when theoretical saturation had been reached.^25^

### 2.4 Participants

Table 1 outlines demographic characteristics of the participants. Of the 23 participants, two were men; thus, women made up much of the sample. Most participants were aged between 35 and 44 and married, with just two one‐parent households represented. Participants held a high level of education, with 18 holding a third‐level degree. All parents had sought unscheduled care for their children in the past for a variety of reasons including falls or injuries, infections, rashes and lethargy, or children refusing food or fluids. The sample had a higher proportion of participants (65%) with private health insurance compared with the current national percentage (46.1%).^26^ Three (13%) participants reported holding a GMS medical card, a proportion much lower when compared to an estimated 32.4% of the population.^27^ The majority of participants had one or two children. None of the participant's children had any current chronic conditions or complex needs. Services accessed included EDs, GPs and OOH GPs.

**Table 1:** Sample Demographics

	n (%)
Gender	
Female	21 (91%)
Male	2 (9%)
Age	
25‐34	5 (22%)
35‐44	17 (74%)
45‐54	1 (4%)
Age of children	
Younger than 2	10 (22%)
2‐5	17 (38%)
6‐12	15 (33%)
13 and older*	3 (7%)
Nationality	
Irish	11 (48%)
European	6 (26%)
African	2 (9%)
Asian	3 (13%)
Other	1 (4%)
Education	
Third level	18 (78%)
Secondary and above	5 (22%)
Health insurance/medical card	
Health insurance	15 (65%)
Medical card	3 (13%)
Neither	5 (22%)
Household status	
One‐parent family	2 (9%)
Two‐parent family	21 (91%)
Location	
Urban (16)	16 (70%)
Rural (7)	7 (30%)

* *While criteria stated they had to be parent to a child younger than 12, some participants also had children older than 12*.

### 2.5 Analysis

**Figure 1:**
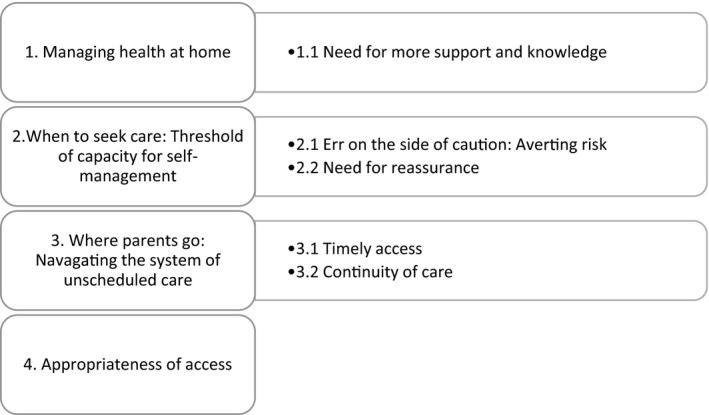
Themes and subthemes

Transcribed data from the focus groups and interviews were thematically analysed concurrently with data collection, informed by a grounded theory approach. ^28^ The data analysis methods were appropriate to apply to both interview and focus group data. Themes and concepts were generated in an inductive manner that involved the researcher going ‘back and forth’ between data and the emerging analysis using comparative methods to generate theories from participants’ narratives in a systematic manner.^28^ Coding consisted of two phases, with initial descriptive coding, sorting and defining themes emerging from the data, followed by axial coding to refine and synthesize the more salient themes under analytical categories.^28^ For example, initial descriptive coding included codes such as ‘identifying and assessing sickness’, ‘parental experience’ and ‘social networks’, which were then subsumed into ‘Managing health at home’ (see Figure 1). Constant comparison methods were applied, whereby cases are compared with one another, as well as with theoretical categories.^29^ This provides a systematic approach to the analytical process, comparing both within and between different cases to organize and develop analytical categories, in order to condense the data.^25^ The analysis was facilitated using NVivo software. 20% of the interviews were independently double‐coded by two researchers (CC and EN), and consensus was reached through discussion on final codes and themes.

### 2.6 Ethics

Research ethics was sought and obtained (LS‐19‐37) from the University Human Research Ethics Committee, and written informed consent was provided before interviews commenced.

## 3 RESULTS

Figure 1 represents the themes and subthemes that developed from the analysis.

### 3.1 Managing health at home

Parents described ways they endeavoured to manage their child's health at home before accessing health care. The extent to which they could do so depended on several circumstantial aspects, for example parental experience, health literacy and social support. Participants referred to being more ‘*reactionary’* [P1] or ‘*scared’* [P19] with first‐born or younger children, due to not having experience, which led them to seek care out of cautiousness. They felt that they gained confidence and skills with experience and could better recognize symptoms, making them more ‘*relaxed’* [P4] and better ‘*equipped’* [P16]. Their health‐care knowledge informed their ability to assess the child's illness and recognize symptoms. Parents strongly valued support from social or family networks, and some participants benefited from informal access to health‐care expertise through friends or family members who were health‐care professionals. In some instances, this resulted in parents feeling reassured enough by the advice and decided against accessing health care, such as in this parent's case:

‘If I didn't have my friend, there's probably a few scenarios where I would have gone to [local OOHs GP] or would have gone to A&E [Accident and Emergency]’ [P16].

Participants discussed their initial reaction to their child falling ill and outlined how they assessed severity through temperature checks and observation of sleeping patterns, and fluid and food intake. This was the first step that informed their decision making regarding seeking care. Parents expressed a preference to manage at home themselves, and often did so if the child's condition was deemed not to require medical attention:

‘I wouldn't be one of these ones that would be running to the doctor every turn around’ [P12].

They described how they adopted a ‘*wait‐and‐see’* [P14] approach, using home remedies or over‐the‐counter medications where possible:

‘I suppose see if we can manage it at home first of all’ [P11].

Some participants referred to their responsibility as a parent to inform and educate themselves on health‐care issues to be able to manage and ensure their child's health:

‘I think that's my own sort of responsibility as a parent to manage the health primarily, then it's the doctor for things like antibiotics, prescription medication things like that’ [P18].

#### 3.1.1 Need for more support and knowledge

Almost all participants expressed a need for more support as to when and where to access care. When discussing instances when they decided to seek care, participants described contacting OOHs services, public health nurses, GPs, private health‐care services, pharmacies or friends with health‐care knowledge to get advice before accessing services. Many mentioned their desire for a helpline to help them make ‘*informed decisions’* [P16]:

‘you don't know really where to look, like who to call or who to go to… []…it's confusing’ [P3].

While difficulty in navigating unscheduled care was expressed by both Irish and non‐Irish participants, migrant communities face particular challenges, such as lack of knowledge of the health‐care system, language barriers, lack of nearby family for support, and differing conceptions of health and expectations of a health system. As unscheduled care is often sought in the context of wanting or needing timely care, such issues can create more stress and anxiety than accessing routine, scheduled care. Non‐Irish participants described grappling with a health system that differed to their home countries, even those with fluent English, married to Irish spouses and residing in Ireland for long periods of time. A few expressed a lack of knowledge of OOHs services, meaning their choice of accessing unscheduled care was limited to a GP or ED, which led to self‐referrals to the ED when GP appointments were unavailable. They felt these services should be better highlighted by their GPs or by authorities:

‘..maybe they give us more information about after hours….I don't know about, I heard I think but I don't really know how it works, if you have to pay’ [P15].

All of this points to insufficient support for parents around accessing unscheduled care for their children.

### 3.2 When to seek care: threshold of capacity for self‐management

When participants reflected on their decision to seek care, and what influenced it, many explained that their assessment of the child's mood and symptoms informed their perception of urgency about their child's condition. Some cases were straightforward, such as broken bones or traumas, in which cases parents self‐referred to the ED. In other instances, the source of the illness was less obvious, and participants described children as lethargic, not taking fluids or eating, or not being themselves as a trigger to seek care. Symptoms that appeared quickly or things they had not experienced before, such as a rash appearing overnight or a temperature ‘*going up like crazy’* [P3], created a sense of uncertainty, which led parents to feel like the situation was beyond their ability to treat at home and to seek care:

‘I suppose scenarios were you just feel completely out of your depth or you know, something you haven't come across before’ [P15].

Parents framed this as a point of capacity, symptoms they could not cope with at home, with one parent describing it as a:

‘threshold…stuff you think you can handle as a parent and stuff you can't’ [P8].

#### 3.2.1 Err on the side of caution: averting risk

Averting risk and the need to avoid any potential harm to their child's health was frequently brought up. Statements such as ‘*Better to be safe than sorry’* [P6] or ‘*err on the side of caution’* [P8] were repeated by participants, who also worried about potential long‐term implications, which prompted them to seek care:

‘the thing as a parent you worry about is this thing that seems small, is it an indicator of something large’ [P8].

One participant mentioned how she balanced her need to ensure her child's well‐being with the necessity of a visit to the doctor, adding additional complexity to the decision to seek care:

‘I suppose I don't wanna look back and say I didn't do something, try and prevent the thing getting worse, kind of erring on the side of caution but balance that with I don't wanna be wasting the doctors time’ [P11].

#### 3.2.2 Need for reassurance

A need for reassurance was often a driver for seeking care; being able to access medical expertise gave parents *‘peace of mind’* [P1] and assured them their child was not in danger. Parents felt medical professionals were best placed to know how serious the illness was, and appreciated advice on recognizing signs of deterioration or easing their child's discomfort. This gave them confidence that they were doing the right thing:

‘it is even that kind of check by a medical person that ‐ no it's OK, you are doing the right thing, you know that means an awful lot’ [P9].

### 3.3 Where parents go: Navigating the System of unscheduled care

#### 3.3.1 Timely access

Once parents felt they had reached the point of capacity to manage at home and decided to access health care, they wanted do so as soon as possible. Parents felt that for children, ‘*quick action’* [P2] was important as their condition can deteriorate quickly. They displayed an unwillingness to wait to access health care:

‘…this is beyond what I think I can do and at that point it's like ok I want the care now, instantly’ [P8].

Timely access to care was crucial in deciding where to access care, and a multitude of factors dictated viable care‐seeking options. Most parents stated their preferred choice for health care was their GP, and therefore, service opening hours and access to their GP was significant. If an appointment was secured in a time frame parents felt was expedient, most opted for this. If they were unable to secure an appointment, they felt they had little option but to seek care elsewhere:

‘if you are looking for an appointment tomorrow and they say no absolutely nothing, you'd be going oh god.. it would push you to go to an OOHs or A&E or somewhere else’ [P11].

Availability of appointments with GPs was crucial in how parents navigated unscheduled care options. Parents varied in their satisfaction with availability; some were able to access GPs when needed, and their practice displayed flexibility in accommodating urgent appointments for their child. Others struggled to secure appointments, and for some, this caused distress. One participant stated how she sometimes felt she had to diagnose the child and ‘*make do’* [P9] with medication available to her. This participant had tried to change GPs previously; however, no other GP in the area had capacity. Another participant relayed her difficulty registering her child with a GP after moving to another county:

‘…I have a little baby here are you telling me if he gets sick I can't take him anywhere’ [P10].

Recipients of GP visit cards or GMS medical cards can only access free care from the GP they are registered with. A couple of participants stated they were willing to pay more to attend another GP practice or private services if it meant quicker access to care:

‘I just paid for a visit cos I couldn't get an appointment in my own practice’ [P1].

For parents who worked full time and dependent on childcare, working hours and health policies implemented by childcare facilities were an additional consideration. If the child fell sick at the weekend, opting for OOHs services was more practical than waiting for a GP appointment on the following Monday as many creches attach conditions to the child's return. For example, some stipulate if the child is taking an antibiotic, they cannot return for at least 48 hours. This illuminates the factors that limit parents’ options when choosing health care, leading them to access higher acuity services in cases when GP services would suffice.

#### 3.3.2 Continuity of care

Participants differed in how much they valued continuity of care. A few were happy with seeing different doctors; one participant brought her children to two different practices. However, most participants did feel continuity was vital in health care, especially for their children. Many tried to maintain continuity, but it was not always an option:

‘We don't really always have that luxury of being able to kind of pick which doctor we want to go to’ [P9].

When it came to accessing care in an unscheduled manner, some parents displayed a willingness to forego continuity in favour of expeditious access to care and see whatever GP had availability when they need it:

‘I have to be more open and go to different doctors cos if my kid is sick I don't want to wait just to be seen in a week just cos I want this doctor, so I just go to any doctor’ [P18].

This further highlights the importance parents placed on timely access to care for their children.

### 3.4 Appropriateness of access

Parents often invoked a sense of social responsibly when it came to utilization of health services and were eager to use them in proportion to the need and urgency of the situation. The ways in which parents framed health‐seeking often echoed literature employing the dichotomy of ‘appropriate’ and ‘inappropriate’ use. Parents voiced reluctance to burden the health system or waste health‐care provider's time and commented on the capacity limitations of services. This may be informed by media depictions of a struggling health system, along with their own experience of long waiting lists for secondary care. One parent referred to the Irish health system as ‘*under‐resourced’* [P15], and others expressed a reticence to ‘*plumb’* [P16] or ‘*clog’* [P12] up the system. Parents juxtaposed their care seeking against others in more need when they explained their decisions not to attend services. One parent spoke about the need to be ‘*responsible citizens’* [P9] and described her uneasiness with utilizing OOH GP services on occasions she could not secure an appointment with the GP:

‘…it doesn't sit well with me because I do get that it's an emergency service and in the back of your head worrying about some old person that might be on their last legs and really need, but at the same time you've got to think of your child, so it's a difficult one’ [P9].

Others echoed this sentiment, with one describing accessing care only when necessarily as the ‘*right thing’* [FGP4], and another referring to guilt in taking capacity from others:

‘I would have felt guilty to be honest that I took a space from someone who was in need’ [P3].

Participants displayed intention to utilize health services in line with the acuity of their children's illness. Some categorized services as ‘*rung[s] of the ladder’* [P11] in terms of urgency, with the ED seen as the most acute. Parents described how they wanted to avoid ‘*jumping the gun’* [P12] and how they have internal conversations or discussions with spouses about the necessity of accessing services, with one explaining how she questioned herself; whether it was ‘*over the top, am I doing too much’* [P6]. This shows how wider discourse around health‐care utilization has permeated parents’ decision making and shaped how they accessed health care for their children.

Some parents moralized health‐seeking behaviour and stressed the validity of their decision in an unprompted manner. In some cases, they emphasized the severity of the symptoms displayed or referring to the diagnoses and medication delivered, which served to justify their health‐seeking. Their consideration of health system capacity was another means to underline this, and they explained how thoroughly they thought about the decision to seek care:

‘Obviously it [going to the ED] wasn't like an impulse you know it was a decision I made based on the symptoms here and like how unresponsive to anything else she was’ [P3].

One participant, who was a medical card holder, distinguished herself against common misconceptions of medical card holders, which centres around the idea that this group takes advantage of free access to health care through unnecessary overuse. This rhetoric made her feel judged by health‐care professionals, and that her health‐seeking was seen as unnecessary and illegitimate:

‘Some people are really trying to do our best at home and then go you know. I don't go just cos I've got a medical card, just go for oh little thing, little scratch or, they cough once oh I'll bring them to the doctor you know, I don't’ [P18].

The fear of judgement was not unique to those with free access to care, and parents who did not hold medical cards also expressed a perceived sense of judgement about using services unnecessarily. They often invoked the stereotypes of ‘*overanxious’* [P6] or ‘*paranoid’* [P4] parents to position themselves against it, to distance themselves from such actions and to justify their health‐seeking behaviour:

‘I know some people, they would, they are so anxious type personalities and everything they would call 999 straight away, I don't want to be like that’ [P2].

In 2015, the government introduced free GP care for children younger than six in Ireland, which a few parents touched upon. While they welcomed and felt fortunate to benefit from the policy, a couple expressed concern that it encouraged overuse, again echoing wider public discourse around the scheme. One participant explained how she was uncomfortable when bringing her children to the GP at first, and felt she was ‘*imposing’ [P4],* due to her perception that her GPs were not properly reimbursed for her visit:

‘it had the opposite effect on me as they claimed it might have on people that they'd be overusing the service. I'd have nearly gone the other way, in so far as I could, because I just felt like I was wasting their time and they weren't getting paid properly for it anyway’ [P4].

This again illuminates the ways in which public discussion around parents’ health‐seeking behaviour permeates parents’ framing of decisions and actions around seeking health care for their children.

## 4 DISCUSSION

This study highlights the multitude of mechanisms that influence how and where parents seek unscheduled care, and the competing considerations parents balance in managing their child's health. While some instances were prompted by the clinical urgency of the illness (ie broken bones), this study highlights how other factors also feed into the decision of when and where parents seek unscheduled care. The results illustrate how parents, to the best of their ability, seek to manage at home; however, their capacity to do so depends on circumstantial factors such as parental experience, health literacy and social support. Participants pointed to the value of support and access to health‐care information and knowledge, through either informal means (family or peers) or formal means (helplines). The decision to seek care was framed as a threshold of capacity, when accessing care parents felt they had reached the limit of what could be managed at home. This was informed by the perceived urgency of the illness, a desire to mitigate risk to their child's health and a need for reassurance. Timely access to medical care was pivotal for parents in deciding what service to attend, largely determined by structural issues such as GP opening hours and availability of appointments. Parents spoke about accessing care in moralistic terms, contextualizing health‐seeking behaviour within public rhetoric of the health system's limited capacity and expressed fear of judgement of how they utilized health services.

The discussion on unscheduled care is often framed around the notion of ‘appropriateness’, although what constitutes as an appropriate or inappropriate visit is perceived differently among health‐care professionals.^30^ Misconceptions about patient behaviour exist, and as this research also highlights, the decision on what service to attend is highly subjective and based on available resources.^7^, ^14^ Reducing ‘inappropriate’ health‐care utilization is a priority for many health systems across the globe; however, factors shaping patterns of access are not accounted for in underlying assumptions of avoidable care.^10^ A study carried out previously shows that parents struggle to navigate services as intended by policymakers, due to the lack of defined boundaries between available services.^5^ This study suggests that the notion of appropriate or inappropriate care seeking has permeated parents’ decision‐making processes and revealed how parents framed their care‐seeking behaviour within this discourse, which resonates with previous research findings.^5^ Furthermore, this study shows how parents were often diverted from general practice to other unscheduled services due to systemic factors such as availability of appointments. The portrayal of health‐seeking as appropriate or inappropriate can lead to delays in seeking care with profound consequences,^31^ an issue that has manifested itself during the recent COVID‐19 pandemic.^32^ This also has implications for health service planning and policy as strategies to reduce ‘inappropriate’ visits are based on discordant or unreliable estimates and assumptions that are not evidence‐based.^7^ Therefore, interrogation of what constitutes appropriate access from different stakeholders’ perspectives is necessary,^14^ and a discursive shift away from framing health‐care access as inappropriate may be warranted.

The ability to navigate unscheduled care options depends on health literacy and local knowledge of the system,^5^ and participants in this study, particularly those from migrant backgrounds, expressed difficulty with this, with a couple holding little or no knowledge of OOH services. Furthermore, all participants reported uncertainty of when they should access care, particularly first‐time parents or those with smaller children. While participants expressed a desire to utilize services in an ‘appropriate’ manner, insufficient knowledge of services available and inaccessible GP care prevented them from doing so. Timely access strongly influenced decision making, and inability to secure a GP appointment in an acceptable time frame often resulted in seeking care elsewhere. Furthermore, this caused distress and frustration for parents. This suggests strengthening access to GP services would better enable parents to manage their children's health and well‐being. This study also illuminates some of the factors that lead to a mismatch between health‐seeking behaviour and intended use of unscheduled health‐care services. More attention needs to be paid to what motivates and shapes care seeking, which can better inform the design of health services.

### 4.1 Implications for policy and practice

These findings point to a need for more resources to educate and support parents. For example, participants identified a desire for a dedicated consultation helpline, currently not available in Ireland. This could help to support capacity to manage at home where possible, and some studies have found high patient satisfaction levels with these services.^33^ The structure of GP care, such as opening hours, that prevents parents from accessing care needs to be examined. Furthermore, undersupply of GPs has previously been identified in Ireland, and demand for GP services is predicted to intensify in the coming years.^34^ This shortage needs to be addressed, along with an exploration as to whether GP services can be delivered in alternative ways, such as nurse‐led services. Finally, co‐design approaches to service design and planning can improve service quality from the perspective of service users,^35^ offer fresh insights to ensure service delivery is responsive to parent and children's health‐care needs and provide an opportunity to develop a common understanding of ‘appropriate’ health‐care use.

### 4.2 Limitations

While this research is situated in a health system that differs from other EU countries in terms of universal access to primary care,^36^ the findings are congruent with studies conducted in other countries with different health systems.^18^ The researcher who carried out the interviews was not a parent, and unfamiliarity of the reality of accessing unscheduled care for children may have impacted on the data collection process. Limitations of the present study also include the relatively homogenous sample. Females made up the majority of the sample, which reflects the gendered nature of studies investigating parental decision making when seeking health care for their children. For example, a recent systematic review examining this issue found that all studies that reported gender were made up of majority female participants.^18^ Further studies should examine whether parental decision making differs by gender. Those educated to third level, living in urban areas and two parent families dominated the sample. Furthermore, those holding medical cards, who are typically higher users of health care, were also underrepresented in this study.

## 5 CONCLUSION

Improved access to GP care and additional supports to parents are required to better facilitate parents in navigating and accessing unscheduled care. Discussions on what constitutes ‘appropriate’ and ‘inappropriate’ access to health care have permeated and shaped parental decision making when accessing unscheduled health care for their children. This framing of health‐seeking needs to be further examined, and a discursive shift away from the concept of ‘appropriate’ or ‘inappropriate’ health‐seeking behaviour may be necessary.

## CONFLICT OF INTEREST

The authors have no conflict of interest to declare.

## AUTHOR CONTRIBUTIONS

EMcA, EN, ADB and TMcD designed the study. CC conducted data collection and prepared the manuscript, with feedback from EN, ADB, TMcD and EMcA. CC and EN carried out analysis. All authors approved the final manuscript.

## INFORMATIVE

This is a qualitative study of parental decision making when accessing unscheduled care for their children.

## Data Availability

The data that support the findings of this study are available from the corresponding author upon reasonable request.

## APPENDIX 1 Topic Guide for Interviews

1. Overall, how do you perceive your child’s current health status? What makes you say 6 this/what informs this judgement?

2. What do when your child is sick?

- Do you feel confident managing your child’s health?
- Can you tell us about a time when you felt you could not manage your child’s health and what you did in that situation?

3. Do you have a regular GP? How long have you been with your GP? How many 18 members of your family attend this GP?

4. Can you talk about your relationship with your GP?

- Can you tell me about any particularly positive experiences you have had with your GP?
- Can you tell me about any particularly negative experiences you have had with your GP?

5. What are your experiences of getting appointments with your GP?

6. Emergency department:

I. Have you ever brought your child to the Emergency Department? (if not, go 35 straight to questions 6(iv) below).

- How many times have you brought your child or children to the ED?

II. Can you talk about a positive experience of bringing your child to the ED?

- How did you make the decision to go to the ED? Did you make the decision alone or was it a joint decision with someone else? What time in the day/day of the week did this occur?
- What did you hope to get from the ED?
- Did you attempt to make contact or get an appointment with your GP beforehand? If so, did the GP refer you to the ED?
- If not, why did you go straight to the ED?
- Is the ED convenient for you to reach?
- Were you satisfied with the care your child received?
- Why do you recall this as a positive experience? Was there a particular incident that occurred that made you recall this experience positively?
- If you were in this situation again, would you make the same choice to go to the ED?

III. Can you talk about a negative experience of bringing your child to the ED?

- Why did you choose to go the ED at this time?
- How did you make the decision to go to the ED? Did you make the decision alone or was it a joint decision with someone else? What time in the day/day of the week did this occur?
- What did you hope to get from the ED?
- Did you attempt to make contact or get an appointment with your GP beforehand?
- Why do you recall this as a negative experience? Was there a particular incident that occurred that made you recall this experience negatively?
- What do you think of the care your child received?
- If you were in this situation again, would you make the same choice to go to the ED?

IV. If you have never brought your child to an ED, has your child ever been very sick?

- What have you done when your child has been very sick?
- Why have you never sought healthcare at an ED?
- In what circumstances do you think you would bring your child to the ED?
- What are your perceptions of the ED?

7. Have you ever used an out‐of‐hours GP service such as Caredoc, Dubdoc etc?

- Can you tell me about this experience?
- Why did you choose the out‐of‐hours service over the ED?

8. Do you find it difficult or easy to access unscheduled services when you need them?

9. Do you feel that these services meet your needs with regards to managing your child’s health?

- If so, what aspects of the service(s) worked well?
- If not, which aspect of the service(s) did not work so well?

10. What changes to these services (if any) would benefit you and your child most?

## APPENDIX 2 Focus group questions and prompts

1. What do you do when your child is sick?

2. Would you consult with others? Do you go to GP/ out of hours/ ED? How do you judge where is best to go?

- What factors are important in establishing a good relationship with GPs?
- Any particularly positive experiences you have had with your GP?
- Any particularly negative experiences you have had with your GP?

3. Emergency department:

- Who has brought thier children to the ED?

4. What makes a positive experience of bringing your child to the ED?

5. What makes a negative experience of bringing your child to the ED?

6. Do you find it difficult or easy to access unscheduled services when you need them?

7. Do you feel that these services meet your needs with regards to managing your child’s health?

- If so, what aspects of the service(s) worked well?
- If not, which aspect of the service(s) did not work so well?
